# Polyclonal origin and hair induction ability of dermal papillae in neonatal and adult mouse back skin

**DOI:** 10.1016/j.ydbio.2012.03.016

**Published:** 2012-06-15

**Authors:** Charlotte A. Collins, Kim B. Jensen, Elizabeth J. MacRae, William Mansfield, Fiona M. Watt

**Affiliations:** a Epidermal Stem Cell Laboratory, Wellcome Trust Centre for Stem Cell Research, University of Cambridge, Tennis Court Road, Cambridge CB2 1QR, UK; b Trangenics Facility, Wellcome Trust Centre for Stem Cell Research, University of Cambridge, Tennis Court Road, Cambridge CB2 1QR, UK; c Epithelial Cell Biology, Cancer Research UK Cambridge Research Institute, Li Ka Shing Centre, Robinson Way, Cambridge CB2 0RE, UK

**Keywords:** Hair follicles, Fibroblasts, Dermal stem cells

## Abstract

Hair follicle development and growth are regulated by Wnt signalling and depend on interactions between epidermal cells and a population of fibroblasts at the base of the follicle, known as the dermal papilla (DP). DP cells have a distinct gene expression signature from non-DP dermal fibroblasts. However, their origins are largely unknown. By generating chimeric mice and performing skin reconstitution assays we show that, irrespective of whether DP form during development, are induced by epidermal Wnt activation in adult skin or assemble from disaggregated cells, they are polyclonal in origin. While fibroblast proliferation is necessary for hair follicle formation in skin reconstitution assays, mitotically inhibited cells readily contribute to DP. Although new hair follicles do not usually develop in adult skin, adult dermal fibroblasts are competent to contribute to DP during hair follicle neogenesis, irrespective of whether they originate from skin in the resting or growth phase of the hair cycle or skin with β-catenin-induced ectopic follicles. We propose that during skin reconstitution fibroblasts may be induced to become DP cells by interactions with hair follicle epidermal cells, rather than being derived from a distinct subpopulation of cells.

## Highlights

► Dermal papillae are polyclonal in origin. ► Hair follicle formation requires fibroblast proliferation. ► Fibroblast proliferation is not required for dermal papilla formation. ► Fibroblasts from skin with inactive Wnt signalling can form dermal papillae.

## Introduction

Mammalian skin consists of a multi-layered interfollicular epidermis with associated hair follicles and sebaceous glands, and an underlying dermis, a collagenous mesenchymal tissue containing fibroblasts and a variety of other cell types, including nerves, blood vessels and immune cells (Millar, 2002; Fuchs, 2008). The dermal papilla (DP) is a cluster of specialized fibroblasts at the base of each follicle, and has a critical inductive role in hair follicle development (Yang and Cotsarelis, 2010; Millar, 2002). DP cells have a number of potential therapeutic applications, both for treatment of hair loss (Jahoda et al., 1984) and as a source of skin-derived progenitors (SKPs) that have the capacity to differentiate into a variety of cell types, including neurons, glia, smooth muscle cells and adipocytes (Toma et al., 2001; Fernandes et al., 2004; Biernaskie et al., 2009).

The mechanisms by which the different dermal subcompartments are constructed remain poorly understood. Hair follicle morphogenesis occurs in successive waves starting from around E14.5 and ending shortly after birth (Millar, 2002; Fuchs, 2008). Fibroblasts are recruited to developing epidermal placodes, where they aggregate to form dermal condensates, and subsequently mature DP, in a process regulated by epidermis-derived Sonic Hedgehog, Platelet-derived growth factor α (PDGFα) and Laminin 511 (Karlsson et al., 1999, St-Jacques et al., 1998; Gao et al., 2008; Millar, 2002). The dermis has multiple embryonic origins, with head and facial fibroblasts being derived from neural crest, and dorsal and ventral trunk fibroblasts originating from somitic and lateral plate dermomyotome, respectively (Driskell et al., 2011). Myf5+/Pax7+ cells in the somitic dermomyotome not only give rise to dorsal dermal fibroblasts but also to skeletal muscle and brown fat (Olivera-Martinez et al., 2004; Lepper and Fan, 2010; Jinno et al., 2010; Driskell et al., 2011). It is unknown whether the unique properties of DP cells reflect a distinct cellular lineage, or are acquired through extrinsic signals from developing hair placodes.

In adult skin, DP can be formed de novo in association with ectopic hair follicles induced by transgenic epidermal activation of β-catenin (Lo Celso et al., 2004; Silva-Vargas et al., 2005) or severe wounding (Ito et al., 2007a). It is unknown whether isolated adult fibroblasts have true hair-initiating capacity outside of these contexts, although Sox2-positive cells in the adult DP and adjacent dermal sheath can support new hair follicle formation and are responsible for dermal maintenance and wound healing (Biernaskie et al., 2009; Driskell et al., 2012). DP cells are normally non-dividing and lineage tracing of adult DP cells suggests that the DP is maintained by cells outside the DP, in the dermal sheath (Chi et al., 2010).

Whether DP are formed and maintained by a specific subset of cells, or via an alternative mechanism, remains unknown. We have explored this question by tracing the origin of DP in chimeric mice and hair follicle reconstitution assays.

## Material and methods

### Mouse strains and chimeras

Experiments were performed in accordance with the UK Government Animals (Scientific Procedures) Act 1986. PdgfraEGFP (PdgfraH2B-eGFP) mice (Hamilton et al., 2003) were obtained from Jackson Laboratories and maintained on a C57 Bl6/ CBA mixed background. The D2 line of K14ΔNβ-cateninER mice was used (Lo Celso et al., 2004). PdgfraEGFP:CAGdsRed, PdgfraEGFP:wild type and PdgfraEGFP:K14ΔNβ-cateninER chimeric mice were generated by aggregation of E2.5 (8 cell) embryos. PdgfraEGFP/K14ΔNβ-cateninER mice and PdgfraEGFP:K14ΔNβ-cateninER chimeras were used at age 6–8 weeks, corresponding to the telogen (resting) phase of the hair cycle.

### Cell isolation

Dissected back skin was scraped free of muscle and fat and incubated overnight at 4 °C in 0.25% Trypsin. The epidermis was then discarded, leaving the majority of DP embedded in the dermis. Neonatal dermis was minced and enzymatically dissociated using Collagenase Type 1 (Sigma) (0.4 mg/mL) for 30 min at 37 °C, with DNase I (20 U/mL) added for the final 5 min. Adult dermis was minced and dissociated for 20 min at 37 °C using a mixture of Collagenase Type I (Invitrogen) (1.25 mg/mL), Collagenase Type II (Worthington) (0.5 mg/mL), Collagenase Type IV (Sigma) (0.5 mg/mL), Hyaluronidase IVS (Sigma) (0.1 mg/mL) and DNase I (50 U/mL). In some experiments, fibroblast proliferation was inhibited by exposing cell suspensions to 50 Gy γ-irradiation in an IBL637 irradiator (CIS Bio International).

### Flow cytometry

Analysis was performed with a CyAn ADP Analyser (Beckman Coulter). Sorting was performed using a MoFlo (DakoCytomation). Cell viability was assessed with a LIVE/DEAD^®^ Viability/Cytotoxicity kit (Invitrogen). In some experiments cells were stained with an APC BrdU Flow Kit (BD Pharmingen) and DAPI staining was used to exclude dead cells. Non-labelled cells and cells labelled with secondary antibody only were used as controls to set gates. Re-analysis of sorted cells in each experiment showed that PdgfraEGFP-positive populations were of 97–100% purity.

### Skin reconstitution

Grafting was performed essentially as described by Lichti et al. (2008) and Jensen et al. (2010). 6 mm diameter chambers (Renner) were inserted into 5 mm diameter punch biopsy wounds on the back skin of 5–8 week old Balb/c nude female mice. Suspensions of keratinocytes and/or dermal cells were injected into the chamber.

### Image acquisition and processing

A Leica SP5 confocal microscope utilising 405, 488 and 561 lasers and Leica Application Suite version 8.2.2 was used. Images were obtained using a 20× HCX PL APO CS dry objective with an L1 405/UV correction optic and a 63× HCX PL APO water-immersion objective with L8 405 and L10 UV correction optics. Images were optimised globally for brightness and contrast using Photoshop CS4 and assembled into figures with Adobe Illustrator CS4.

## Results

### Polyclonal origin of DP in neonatal skin

**Fig. 1:**
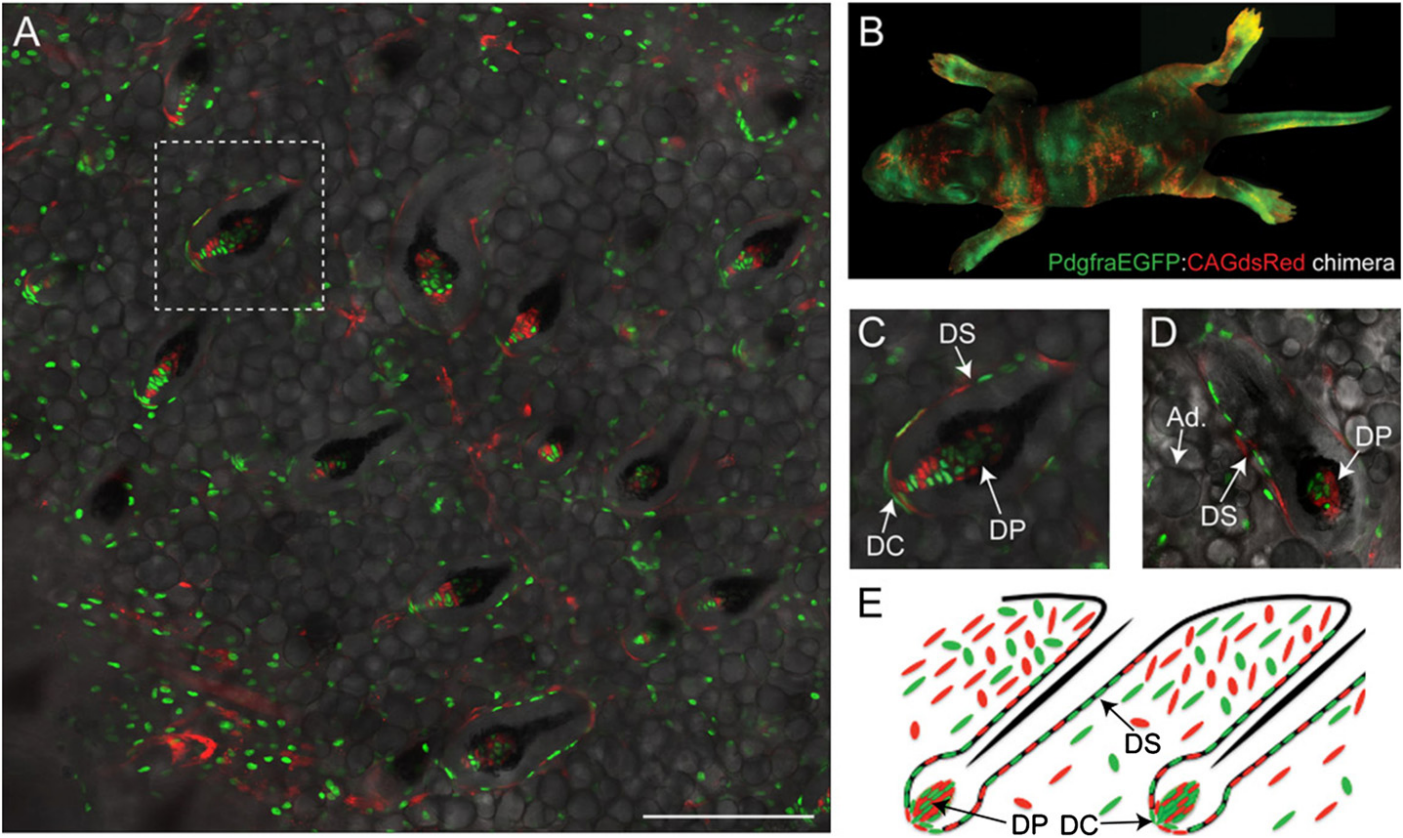
DP formation in chimeric mice. (A, C, D) Wholemounts of PdgfraEGFP:CAGdsRed chimeric P2 neonatal back skin. Images of endogenous GFP (green) and dsRed (red) fluorescence overlaid onto brightfield images of the same field. C is an enlarged region of A. (B) Composite image of PdgfraEGFP:CAGdsRed chimeric P4 neonate. (E) Schematic depiction of the composition of different dermal subcompartments. DP: dermal papilla; DS: dermal sheath; DC: dermal cup. Scale bars: 200 μm. (For interpretation of the references to colour in this figure legend, the reader is referred to the web version of this article.)

For non-biased lineage analysis of the DP, we generated chimeric mice by in vitro aggregation of E2.5 blastocysts derived from two different fluorescent mouse strains. CAGdsRed transgenic mice express dsRed in all tissues via the chicken actin promoter. PdgfraEGFP mice (PdgfraH2BeGFP, Hamilton et al., 2003) express a stable, nuclear form of GFP from the endogenous Pdgfra promoter. In PdgfraEGFP skin, GFP is expressed by all dermal fibroblasts but is absent from other cell populations, including keratinocytes, melanocytes, blood and endothelial cells, as judged by flow cytometry and Q-PCR for markers of each cell type (Collins et al., 2011). PdgfraEGFP:CAGdsRed aggregate embryos were implanted into pseudo-pregnant females and the resultant viable neonates were viewed under a fluorescence dissection microscope. Seven neonates exhibiting patchy contribution of GFP and dsRed were analysed (Fig. 1).

Wholemounts of neonatal back skin were imaged from the dermal side. We examined obvious regions of microchimerism that contained similar contributions of the two colours (Fig. 1A and B). In these regions, 100% of DP in skin (>50 DP analysed per mouse) of 7/7 mice contained a mixture of PdgfraEGFP-positive and CAGdsRed-positive cells. The dermal cup (fibroblasts at the lowermost base of the hair follicle, beneath the dermal papilla) and dermal sheath (DS) (fibroblasts surrounding the hair follicle) were also typically composed of a mixture of GFP-positive and dsRed-positive cells, indicating that they too are polyclonal (Fig. 1A, C–E).

### Polyclonal origin of ectopic DP induced in adult skin

**Fig. 2:**
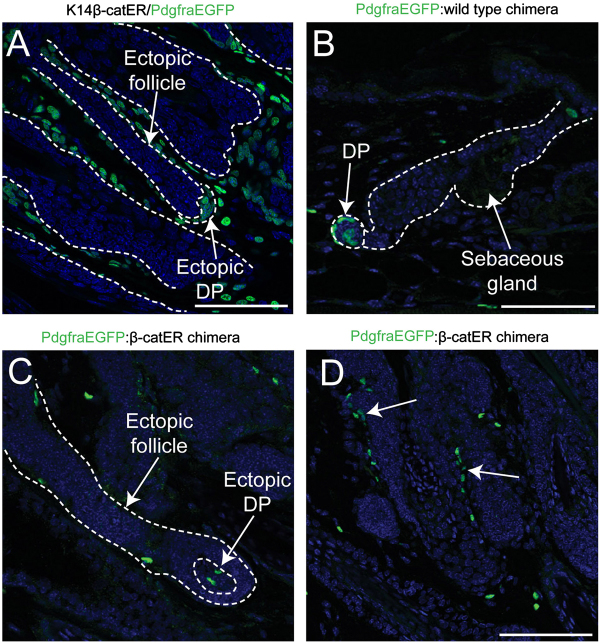
Polyclonal origin of DP associated with β-catenin induced adult ectopic hair follicles. (A–D) Paraffin sections of adult 4OHT-treated back skin from (A) PdgfraEGFP/K14ΔNβ-cateninER heterozygous mouse; (B) PdgfraEGFP:wild type chimeric mouse; (C, D) PdgfraEGFP:K14ΔNβ-cateninER chimeric mice. Anti-GFP: green; DAPI: blue. Arrows in (D) point to GFP-positive cells emanating from the hair follicle junctional zone. Scale bars=100 μm. (For interpretation of the references to colour in this figure legend, the reader is referred to the web version of this article.)

New hair follicles normally form only during late embryonic and early neonatal development. However, in K14ΔNβ-cateninER mice, new hair follicles can be generated in adult skin in response to 4-hydroxytamoxifen (4-OHT)-induced epidermal activation of β-catenin. Ectopic follicles encapsulate a DP-like structure that expresses alkaline phosphatase and other DP markers (Lo Celso et al., 2004; Silva-Vargas et al., 2005) but is Sox2-negative (Driskell et al., 2012). The DP gene signatures of fibroblasts from telogen, anagen and neonatal skin and adult skin with ectopic follicles are all distinct (Collins et al., 2011). As expected (Collins et al., 2011), when K14ΔNβ-cateninER and PdgfraEGFP mice were crossed to generate double heterozygous animals, all DP cells and all other dermal fibroblasts were positive for PdgfraEGFP (Fig. 2A). In control chimeras of PdgfraEGFP and wild type mice, many DP contained both GFP-positive and -negative cells (Fig. 2B), indicating that DP remain polyclonal in adulthood.

To determine whether the DP of β-catenin-induced ectopic follicles were polyclonal, we generated K14ΔNβ-cateninER:PdgfraEGFP chimeras, in which GFP-negative fibroblasts are contributed by the K14ΔNβ-cateninER genotype. 4-OHT treatment of back skin resulted in patchy hair re-growth in 4/8 chimeras, consistent with the existence of regions of β-catenin activation (derived from the K14ΔNβ-cateninER embryo) and non-activation (derived from the PdgfraEGFP embryo) (not shown). The DP of the original adult hair follicles (both telogen and anagen) contained both GFP-positive and GFP-negative cells (data not shown). Where regions of K14β-cateninER-positive epidermis were juxtaposed to regions of PdgfraEGFP-positive dermis, we also examined the DP of ectopic follicles (*n*=5–10 per mouse). They were frequently formed from mixtures of GFP-positive and GFP-negative cells, indicating that they too were polyclonal (Fig. 2C).

Examination of areas of K1414ΔNβ-cateninER:PdgfraEGFP chimeric back dermis with a low contribution of PdgfraEGFP-positive cells (estimated 5–20%) revealed an absence of GFP-positive patches containing more than two or three cells, implying limited proliferation or rapid dispersal of progeny following cell division. However, larger groups of GFP-positive cells (possibly clones) were sometimes seen adjacent to the hair follicles (Fig. 2D). It is possible that these cells are associated with the hair follicle junctional zone, the location of Lrig1-positive epidermal stem cells, since fibroblasts adjacent to the junctional zone proliferate and accumulate in response to epidermal activation of β-catenin (Collins et al., 2011).

Our results demonstrate a polyclonal origin of the DP during skin development, in adulthood and when ectopic follicles are induced in adult skin by epidermal β-catenin activation.

### Hair follicle neogenesis requires fibroblast proliferation

**Fig. 3:**
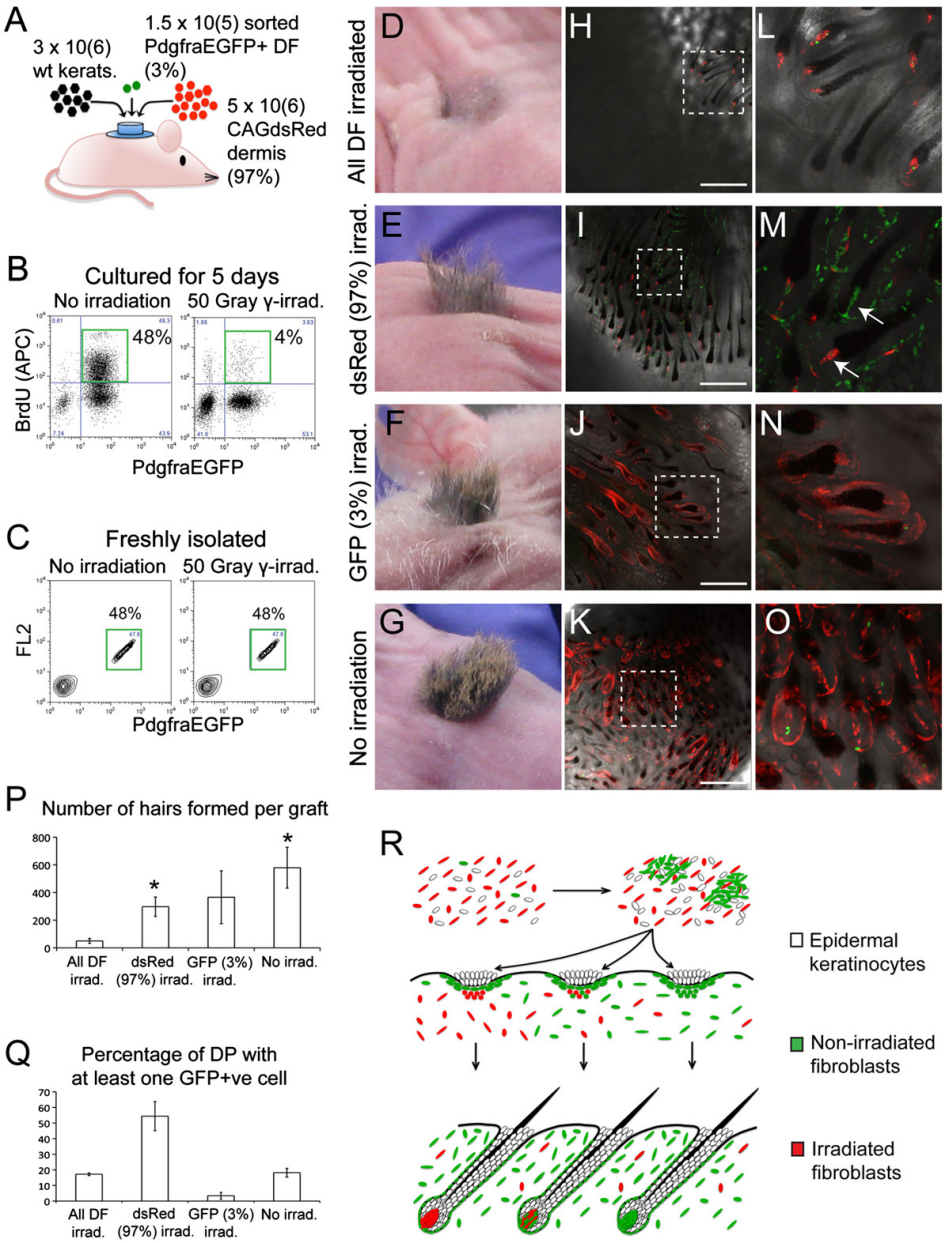
Irradiated neonatal fibroblasts give rise to DP but not DS. (A) Experimental set-up. (B) GFP labelling and BrdU incorporation in non-irradiated and irradiated dermal cells after 5 day in culture. (C) Purification of viable GFP-positive cells from non-irradiated and irradiated PdgfraEGFP dermis. Cells were examined 1 h after isolation. (D–G) Hair growth at graft sites. (H–O) Grafts viewed from the dermal side. Endogenous GFP (green) and dsRed (red) fluorescence overlaid onto brightfield images of the same fields. L–O are enlargements of selected regions of H–K, respectively. Arrows in M point to graft-derived DP that are either entirely GFP-positive or entirely dsRed-positive. Note GFP-positive DS in each case. Scale bars: 200 μm. (P) Mean number of hairs±SEM formed per graft. (Q) Mean percentage (± SEM) of graft-derived DP containing one or more GFP-positive cells (*n*=3 grafts/group; 43–118 follicles scored/ graft). (P, Q) Asterisks denote a significant difference from the ‘All DF irrad.’ group (T test, *p*<0.05). (R) Schematic representation of the results. (For interpretation of the references to colour in this figure legend, the reader is referred to the web version of this article.)

Cells within DP rarely divide (Tobin et al., 2003) and the polyclonal origin of DP suggests DP formation may not be dependent on proliferation. To test this, we used skin reconstitution assays. An inert protective chamber was implanted into a full thickness wound on the back of a nude mouse and disaggregated neonatal fibroblasts and epidermal cells (predominantly keratinocytes with some melanocytes also present) were injected into the chamber, resulting in formation of hair-bearing skin within 3–5 weeks (Lichti et al., 2008; Jensen et al., 2010; Fig. 3A). To inhibit proliferation, we exposed freshly-isolated fibroblasts to 50 Gy γ-irradiation. When cells were subsequently placed in culture and monitored for 5 day after plating, there was no effect of irradiation on cell viability, but cell division was inhibited (Fig. 3B).

To assess the relative hair-initiating capacity of irradiated and non-irradiated fibroblasts, we used mixtures of dermal cells expressing different fluorescent markers. Each graft (*n*=3/group) consisted of 3×10^6^ wild type neonatal epidermal cells, 5×10^6^ CAGdsRed non-fractionated neonatal dermal cells (97% of total dermal cells) and 1.5×10^5^ sorted PdgfraEGFP-positive neonatal dermal fibroblasts (3% of total dermal cells) (Fig. 3A). Non-fractionated dermal cells contain between 45% and 70% fibroblasts (PdgfraEGFP-positive) (Fig. 3C). Each graft contained the same number and proportion of fibroblasts, with the only experimental variable being whether or not fibroblasts had been irradiated. We used four experimental groups (*n*=3 grafts/group), in which: (1) both dermal components were irradiated (Fig. 3D,H,L); (2) only the CAGdsRed cells (97% of dermal cells) were irradiated (Fig. 3E,I,M); (3) only the PdgfraEGFP fibroblasts (3% of dermal cells) were irradiated (Fig. 3F,J,N); or (4) neither fraction was irradiated (Fig. 3G, K, O).

After four weeks, grafts in which neither dermal fraction was irradiated generated 579±148 hairs. Grafts in which both dermal fractions were irradiated gave rise to 11 fold fewer hairs (average 50±18; T test *p*<0.05), demonstrating that hair follicle neogenesis required fibroblast proliferation. Nevertheless, grafts in which only the CAGdsRed fraction (97%) was irradiated formed an average of 297±70 hairs, a 6-fold increase over grafts in which 100% of dermal cells were irradiated (*p*<0.05). Therefore the 3% PdgfraEGFP-positive fraction of proliferation-competent cells was remarkably efficient at restoring hair-initiation ability to irradiation-depleted dermis (Fig. 3D–G, P; Table 1).

### DP can form from mitotically inhibited cells

To estimate the contribution of GFP-positive cells to DP formation we used confocal microscopy to examine grafts as wholemount preparations from the dermal surface (Fig. 3H–O). The dsRed signal was well preserved in the wholemounts and so individual cells could be scored unambiguously as GFP+ or dsRed+. We scanned several confocal planes of each DP and then scored whether all the cells observed were GFP+ (100%) or none (0%). In the case of DP containing both GFP+ and dsRed+ cells the GFP+ contribution was estimated as being greater or less than 50% (Table 1).

The percentages of DP containing at least one GFP-positive cell are shown in Fig. 3Q. Grafts in which neither dermal fraction was irradiated, or in which both dermal fractions were irradiated, contained similar percentages of DP with one or more GFP-positive cell (18.2±0.9% and 17.2±2.8%, respectively), consistent in both cases with neither the CAGdsRed nor the PdgfraEGFP-positive cells having a selective advantage. In grafts where only the PdgfraEGFP positive fraction (3%) was irradiated, only 3.4±2.2% of DP contained one or more GFP-positive cell. However, in grafts where only the CAGdsRed fraction (97%) was irradiated, 54.4±9.4% of DP contained one or more GFP-positive cell, indicating a substantial expansion of the GFP-positive population (Fig. 3H–O,Q).

In grafts in which only the CAGdsRed fraction (97%) was irradiated, approximately 44% of DP (72/162) were composed entirely of CAGdsRed-positive irradiated cells, showing that mitotically inhibited fibroblasts remained capable of forming DP (Fig. 3I–M,Q Table 1). Nevertheless, the dermal sheaths of follicles in these grafts were almost entirely derived from the 3% of proliferation-competent GFP-positive cells, and not from the irradiated CAGdsRed cells (Fig. 3I,M).

We conclude that fibroblast proliferation is a necessary step in hair follicle morphogenesis, most likely because it is required for formation of the dermal sheath (Chi et al., 2010). However, DP can form from mitotically inhibited cells (Fig. 3R).

### Adult dermal fibroblasts retain the ability to initiate hair follicle neogenesis

Hair follicle neogenesis normally occurs only during the late embryonic and early neonatal phases of development (Millar, 2002; Fuchs, 2008). However, adult epidermal keratinocytes reveal an excellent ability to generate new hair follicles when combined with neonatal fibroblasts in graft assays (Lichti et al., 2008; Yang and Cotsarelis, 2010). Since the number of DP cells and their gene expression profiles differ between different hair cycle stages (Tobin et al., 2003; Chi et al., 2010; Greco et al., 2008; Collins et al., 2011), the absence of hair follicle neogenesis in adult skin could be due to intrinsic differences in the inductive properties of adult and neonatal fibroblasts. Alternatively it could reflect the decline in fibroblast density that occurs postnatally (Collins et al., 2011). To distinguish between these possibilities, we designed a skin reconstitution experiment to compare the hair-initiating capacity of equal numbers of PdgfraEGFP-positive fibroblasts isolated from skin in different developmental states: adult telogen, adult anagen (K14ΔNβ-cateninER mice treated once with 4OHT to induce anagen), adult with anagen and ectopic follicles (K14ΔNβ-cateninER that received repeated applications of 4OHT), and neonatal.

**Fig. 4:**
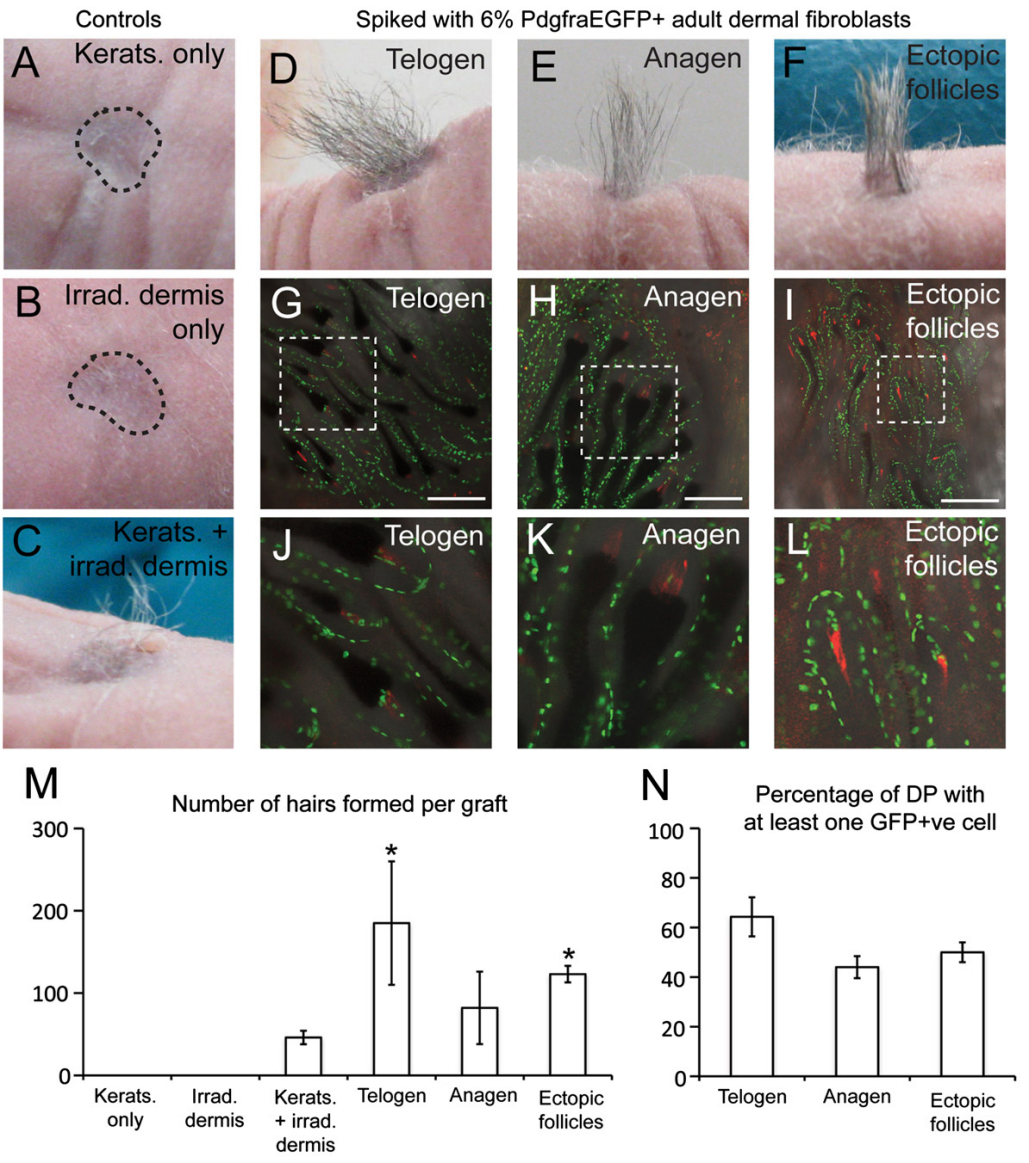
Adult dermal fibroblasts initiate hair follicle formation in skin reconstitution assays. (A–F) Hair growth at graft sites. (G–L) Grafts viewed from the dermal side. Endogenous GFP (green) and dsRed (red) fluorescence is overlaid onto brightfield images of the same fields. (J–L) Enlargements of selected regions of (G–I), in respective order. (M) Average number of hairs (± SEM) formed per graft. (N) Mean % (± SEM) graft-derived DP with one or more GFP-positive cell (*n*=3–4 grafts/group; 15–31 follicles scored/graft). Asterisks denote a significant difference from the ‘Kerats.+irrad. dermis’ group (T test, *p*<0.05). Scale bars: 200 μm. (For interpretation of the references to colour in this figure legend, the reader is referred to the web version of this article.)

Only small numbers of fibroblasts can be purified from adult skin. However, our previous experiment showed that the hair-initiating ability of irradiated neonatal dermis could be restored by the addition of a small number of proliferation-competent fibroblasts (Fig. 3E, I–M,P). For each graft, we therefore used 5×10^6^ irradiated, non-fractionated CAGdsRed neonatal dermal cells spiked with 3×10^5^ purified PdgfraEGFP-positive fibroblasts (6% of total dermal cells), combined with 4×10^6^ adult wild type keratinocytes (Fig. 4).

Grafts of epidermal cells alone (*n*=2) or irradiated fibroblasts alone (*n*=2) did not result in hair growth (Fig. 4A and B,M; Table 2). Grafts of keratinocytes combined with irradiated CAGdsRed fibroblasts only (*n*=3; non-spiked control) generated an average of 46±8 hairs (Fig. 4C,M; Table 2). However, addition of a spike of 6% PdgfraEGFP-positive cells (*n*=3 or 4 grafts per group) resulted in a 2 to 4-fold increase in the number of hairs generated, irrespective of whether the cells originated from adult telogen, adult anagen, adult ectopic follicle-forming or neonatal dermis (Fig. 4D–F, M; Table 2). The increases in hair number were statistically significant for the telogen and ectopic follicles groups when compared to the non-spiked control (T test *p*<0.05 in each case; Fig. 4M).

Our results show that, as with neonatal fibroblasts, a small number of adult fibroblasts can restore hair-initiating capacity to irradiated dermis. We conclude that adult fibroblasts retain the capacity to contribute to DP formation.

## Discussion

Our studies have revealed that mouse back skin DP are polyclonal, regardless of whether they are present in neonatal or adult skin or are formed in association with β-catenin-induced adult ectopic follicles. Although fibroblast proliferation is necessary for hair follicle formation in skin reconstitution assays, the cells that form the DP can be mitotically inactive. This, together with the inverse relationship between DP cell expansion in hydrogel culture and hair inducing ability in grafts (Driskell et al., 2012), suggests that the hair growth induction efficiency of DP cells is due to their morphogenetic, rather than proliferative, capacity. We also found that fibroblasts from neonatal, telogen, anagen and anagen with ectopic follicles skin are all competent to contribute to DP formation. The extent to which our findings are applicable to human hair follicles remains to be determined.

The finding that DP cell proliferation is not required for hair follicle formation is consistent with earlier evidence that the dermal sheath is the source of DP cells (Tobin et al., 2003; McElwee et al., 2003; Chi et al., 2010; Horne and Jahoda, 1992; Yamao et al., 2010). Although DP cells do have unique properties, such as spontaneous aggregation in culture (Jahoda and Oliver, 1984) and hair initiation capacity (Ito et al., 2007b), those properties can be conferred on DS cells through exposure to signals from epidermal cells in the hair follicle matrix (Reynolds and Jahoda, 1996).

Since β-catenin induced ectopic follicles have associated DP (Silva-Vargas et al., 2005), we had anticipated that sustained epidermal β-catenin activation would increase the competence of the underlying dermis to support ectopic follicle formation; however, that was not the case. We found that adult telogen and anagen fibroblasts could contribute to the DP of new hair follicles, even though the competence of DP cells to induce follicles has been reported to vary during the hair growth cycle (Iida et al., 2007) and follicle neogenesis is normally restricted to embryonic and neonatal skin. Since the absence of hair follicle neogenesis in normal adult skin does not reflect loss of intrinsic developmental potential, it must be due to other factors, such as low fibroblast density or mitotic quiescence (Collins et al., 2011).

Our studies do not, however, rule out the existence of different fibroblast subpopulations. For example, we have observed that fibroblasts associated with the hair follicle junctional zone are particularly sensitive to the proliferative stimulus associated with β-catenin-induced ectopic follicle formation (Collins et al., 2011). Although lineage tracing has shown that postnatal Corin-positive DP cells do not contribute to other dermal compartments (Enshell-Seijffers et al., 2010), it has been reported that Sox2-positive cells of the DP and dermal sheath are responsible for dermal regeneration following wounding of adult skin (Biernaskie et al., 2009). We have also found intrinsic differences in the proliferative potential and dermal contribution of Sox2-positive and –negative DP cells following expansion in culture (Driskell et al., 2012). Nevertheless, Sox2+ cells are unlikely to make a significant contribution to the DP in the present experiments. Sox2 is not expressed in the DP of zigzag hair follicles, which are the major hair type in postnatal back skin (Driskell et al., 2009), or the DP of β-catenin-induced ectopic follicles (Driskell et al., 2012). In skin reconstitution assays, as few as 3% proliferation-competent fibroblasts could restore the hair-initiation potential of an irradiated dermal niche, which is reminiscent of the behaviour of stem cells in blood (Kondo et al., 2003) and skeletal muscle (Zammit, 2008). Therefore, it is of considerable interest to pursue the issue of dermal cell heterogeneity further.

## Figures and Tables

**Table 1:** % contribution of neonatal PdgfraEGFP-positive cells to individual graft-derived dermal papillae. Graft sites were viewed as wholemounts from the dermal side to visualise graft-derived DP. In three 20× fields per graft, each DP was examined in several focal planes. If all the cells observed were GFP+ the DP was scored as 100% GFP+ and if all the cells were dsRed+ the DP was scored as 0% GFP+. In the case of DP containing both GFP+ and dsRed+ cells, the contribution of GFP+ cells was estimated as greater or less than 50%. DF: dermal fibroblasts.

**Group**	**Graft**	**Total hairs**	**100% GFP**	**>50% GFP**	**<50% GFP**	**0% GFP**	**DP scored**
**All DF irradiated**	**1**	21	0	0	8	35	43
	**2**	71	0	0	8	41	49
	**3**	58	0	0	11	55	66
**CAGdsRed (97%) irradiated**	**1**	408	5	15	23	21	64
	**2**	265	4	9	14	22	49
	**3**	217	3	6	11	29	49
**PdgfraEGFP (3%) irradiated**	**1**	54	0	0	2	45	47
	**2**	537	0	0	0	40	40
	**3**	507	0	0	3	48	51
**No irradiation**	**1**	376	0	0	22	96	118
	**2**	567	0	0	11	39	50
	**3**	794	0	0	7	43	50

**Table 2:** % contribution of PdgfraEGFP-positive cells to individual graft-derived dermal papillae. Graft sites were viewed and scored as for Table 1. DF: dermal fibroblasts.

**Group**	**Graft**	**Total hairs**	**100% GFP**	**>50% GFP**	**<50% GFP**	**0% GFP**	**Total counted**
**Kerats. only**	**1**	0	–	–	–	–	–
	**2**	0	–	–	–	–	–
**Irrad. dsRed DF only**	**1**	0	–	–	–	–	–
	**2**	0	–	–	–	–	–
**Kerats. + Irrad. dsRed DF only**	**1**	37	–	–	–	–	–
	**2**	42	–	–	–	–	–
	**3**	59	–	–	–	–	–
**6% Telogen PdgfraEGFP**	**1**	82	3	2	15	6	26
	**2**	293	0	1	8	6	15
	**3**	181	0	2	12	11	25
**6% Anagen PdgfraEGFP**	**1**	61	0	0	7	7	14
	**2**	151	0	0	8	10	18
	**3**	32	0	0	6	10	16
**6% Ectopic follicle PdgfraEGFP**	**1**	107	1	2	10	9	22
	**2**	113	0	1	10	12	23
	**3**	125	0	3	12	16	31
	**4**	145	0	2	7	12	21
**6% Neonatal PdgfraEGFP**	**1**	457	6	19	17	9	51
	**2**	91	1	3	7	3	14
	**3**	40	2	4	9	6	21
